# Environmental and Pharmacological Manipulations Blunt the Stress Response of Zebrafish in a Similar Manner

**DOI:** 10.1038/srep28986

**Published:** 2016-06-28

**Authors:** Ana Cristina V. V. Giacomini, Murilo S. Abreu, Rodrigo Zanandrea, Natália Saibt, Maria Tereza Friedrich, Gessi Koakoski, Darlan Gusso, Angelo L. Piato, Leonardo J. G. Barcellos

**Affiliations:** 1 Programa de Pós-Graduação em Farmacologia, Universidade Federal de Santa Maria (UFSM), Av. Roraima, 1000, Cidade Universitária, Camobi, Santa Maria, RS 97105-900, Brazil; 2 Universidade de Passo Fundo (UPF), BR 285, São José, Passo Fundo, RS 99052-900, Brazil; 3 Programa de Pós-Graduação em Farmacologia e Terapêutica, Instituto de Ciências Básicas da Saúde, Universidade Federal do Rio Grande do Sul, Porto Alegre, RS 90050-170, Brasil; 4 Programa de Pós-Graduação em Bioexperimentação, Universidade de Passo Fundo(UPF), BR 285, São José, Passo Fundo, RS 99052-900, Brazil

## Abstract

Here we provide evidence that both pharmacological and environmental manipulations similarly blunt the cortisol release in response to an acute stressor in adult zebrafish. Different groups of fish were maintained isolated or group-housed in barren or enriched tanks, and then exposed or not to diazepam or fluoxetine. Acute stress increased cortisol levels in group-housed zebrafish maintained in barren environment. Single-housed zebrafish displayed a blunted cortisol response to stress. Environmental enrichment also blunted the stress response and this was observed in both isolated and group-housed fish. The same blunting effect was observed in zebrafish exposed to diazepam or fluoxetine. We highlighted environmental enrichment as an alternative and/or complimentary therapeutic for reducing stress and as a promoter of animal welfare.

According to the regulatory agencies of research procedures and animal welfare^1^, experimental animals must be housed in environments with space and complexity to allow their normal behavioral expression. Thus, several studies have been conducted using environmental enrichment in behavioral experiments in rats^2^,^3^, mice^4^,^5^, humans^6^ and fish^7^,^8^.

One of the most used fish models is the teleost zebrafish (*Danio rerio*). Zebrafish have been used in several research areas due to genetic homology with humans^9^, as a model organism in neuroscience and behavioral studies, as well as to test candidate drugs^10^,^11^,^12^. The effects of many psychiatric drugs have been widely studied in relation to the zebrafish stress response^13^,^14^,^15^.

Zebrafish exhibit social behavior since early stages of life^16^, preferring swimming in shoals^17^. In fact, behavioral and endocrine responses may differ according to the housing conditions and thus may be particularly influenced by group and/or isolation^18^,^19^,^20^, as well as environment complexity.

However, to our knowledge, there are no studies regarding the effects of environmental enrichment, psychotropics and their association (environmental enrichment plus psychotropics) in different housing conditions on the zebrafish stress response. Hence the question: do environmental complexity and psychotropics modulate the stress neuroendocrine axis in different fish housing conditions? To answer this question, our strategy was to evaluate the acute stress response in single- or group-housed fish maintained in environmentally enriched or barren tanks, and exposed or not to diazepam or fluoxetine for a 15-day period.

## Results

### Waterborne concentrations of FLU and DZP

**Figure 1:**
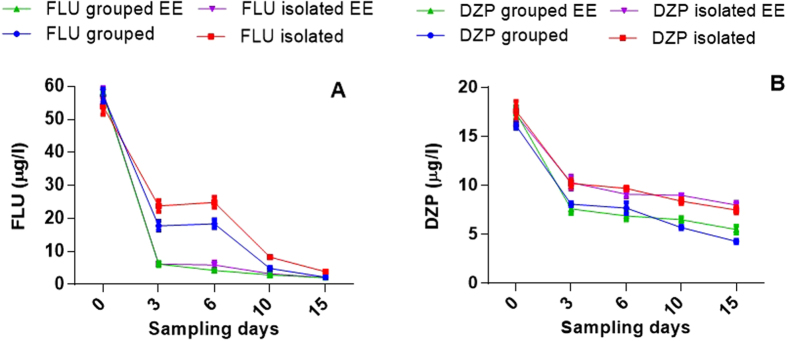
Time course of FLU (A) and DZP (B) degradation during 15-day period. EE—environmental enrichment. Data were expressed as mean ± S.D. of three water samples in which time point.

Concentrations of FLU and DZP declined at 15 days after exposure compared to the nominal concentration. They remained above the range of ecologically relevant concentrations. Neither FLU nor DZP were detected in control water samples (Fig. 1A,B).

### Housing condition

**Figure 2:**
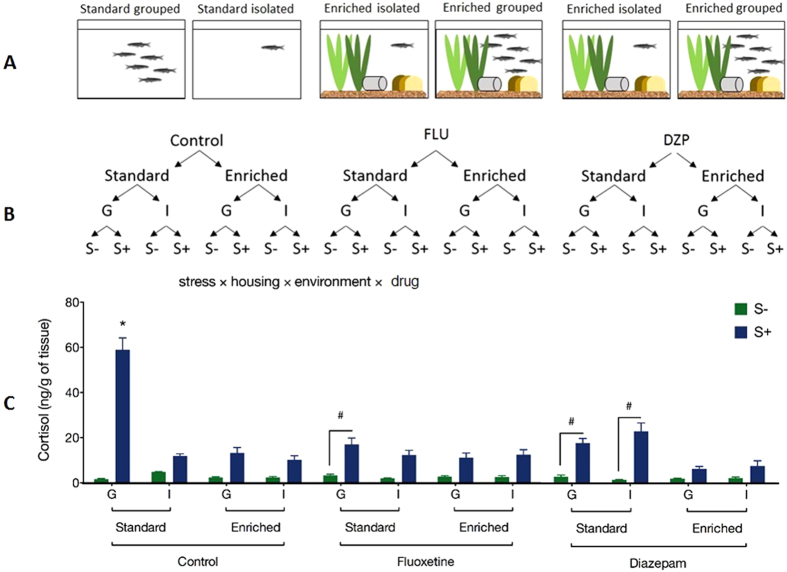
Study design and results. (**A**) Aquaria setup; (**B**) schematic representation of the experimental design; Effects of acute stress on whole-body cortisol levels in zebrafish housed in groups (G) or isolated (I) in standard or enriched tanks and exposed or not to fluoxetine or diazepam (**C**). Four-way ANOVA followed by Tukey’s multiple comparison test. *p < 0.05 compared to all others groups; ^#^p < 0.05 compared to the depicted groups. The drawing in the panel A were drawn by LB.

The basal cortisol levels were similar in all groups. Acute stress increased cortisol levels in group-housed zebrafish maintained in standard environment (barren tanks). Single-housed zebrafish displayed a blunted cortisol response to stress (Fig. 2C,D).

### Environmental enrichment

Environmental enrichment blunted the cortisol response to stress in both isolated and group-housed fish. The same effect was observed in zebrafish exposed to diazepam or fluoxetine (Fig. 2C,D).

### Drug exposure

Both diazepam and fluoxetine exposure blunted the cortisol response to stress in isolated and group-housed fish. The effects of diazepam in blunting the cortisol response were greater when combined with environmental enrichment (Fig. 2C,D). The 4-way ANOVA yielded significant main effects and interaction effects for housing, environment, stress and drug. Statistical data is shown in supplementary materials.

## Discussion

Here we provide evidence that both pharmacological and environmental manipulations similarly blunt the cortisol release in response to an acute stressor in adult zebrafish.

We achieve this conclusion since fish housed in environmental enriched tanks as well those exposed to fluoxetine or diazepam presented lower cortisol concentrations than fish housed in barren tanks. We also show that the environmental enrichment is capable to abolish the difference in cortisol concentrations between isolated and grouped zebrafish housed in barren tanks verified in our previous work^19^. Furthermore, cortisol levels after acute stress in fish housed in environmental enriched tanks are similar to unstressed fish.

We hypothesized that the stress-blunting effect of environmental enrichment occurs by providing fish a sense of safety or security in a natural environment with refuge alternatives. The difference between barren versus enriched is in the context in which the fish are housed, as the enriched environment is a natural environment that offers wellness with plants, sand and stones that can serve as protection from threats and interaction; while the barren aquarium does not meet these conditions making fish more vulnerable and therefore with a more responsive stress response – they remain on alert state.

In addition, after 15 days, the response to acute stress observed in fish housed in an enriched environment is similar to fish exposed to diazepam or fluoxetine. Although the inoculation of these drugs was made at the begin of the experimental period, the concentrations of fluoxetine measured at the end of the exposure period are sufficient to block the cortisol response to acute stress in zebrafish^13^. The lower concentrations measured reflect a combined effect of uptake by the fish^21^,^22^,^23^,^24^, adsorption to organic matter and photodegradation^25^,^26^. In the end of the exposure on day 15, drug concentrations remained above the range of ecological relevance^27^,^28^,^29^,^30^. Regarding DZP here show that the concentration at the end of exposure period blocked the response to acute stress. The decrease in the stress response by pharmacological agents (diazepam and fluoxetine) may represent the fish being susceptible to a possible lack of response when its needed, as the ability to promote ionic^31^, metabolic^32^ and behavioral^15^ necessary adjustments of the stress response.

Acute stress increases anxiety-like behavior, reduces social interaction and increases aggression in zebrafish that are modulated by fluoxetine and diazepam^15^. The similarity between the effect caused by environmental enrichment and exposure to drugs is thus reinforced by behavioral studies^2^,^3^,^4^,^5^,^7^.

The anxiolytic effect of environmental enrichment has been demonstrated in different species^2^,^3^,^4^,^5^,^7^. The preference by interaction with conspecific is abolished in fish isolated in enriched environment^8^, and in fish submitted to the acute exposure to fluoxetine and diazepam^15^.

Some studies show relationship between environmental enrichment and serotonin. In fact, environmental enrichment increases the expression of 5-HT receptors in mice^5^ and increase in threshold of pain^33^,^34^.

The environmental manipulation can be seen as a non-pharmacological approach to reduce the withdrawal symptoms^2^,^35^ and depressive-like behavior in rats^2^,^5^. In addition, environmental enrichment restores serum corticosterone and BDNF levels^4^,^36^ in the hippocampus of mice with Rett syndrome^4^ and rats submitted the prenatal morphine exposure^36^. In humans, environmental enrichment might be considered as a complementary treatment for autism^6^,^37^ and affective disorders and anxiety^3^,^4^,^5^,^36^.

Given the translational importance of zebrafish, we highlighted environmental enrichment as an alternative and/or complimentary therapeutic for reducing stress and as a promoter of animal welfare.

## Methods

### Fish

A stock population of 372 mixed-sex (50/50) adult wild-type zebrafish (*Danio rerio*), short-fin (SF) strain, was randomly distributed, isolated or in kept in groups of six fish in barren or enriched tanks and exposed for 15 days to diazepam or fluoxetine. A control group was submitted to the same experimental conditions but without pharmacological treatment (see the scheme in Fig. 2B).

This experimental setup was approved by the Ethics Commission for Animal Use of the Universidade de Passo Fundo, Brazil (Protocol *#*09/2014) and followed the guidelines of the Conselho Nacional de Controle de Experimentação Animal (CONCEA).

### Drug exposure

We used fluoxetine (Daforin®, EMS, Brazil) and diazepam (União Química, Brazil) at 50 μg/L and 16 μg/L, respectively based on previous results from^13^. We exposed fish to these drugs for 15 days. This period is considered a sufficient time to elicit different responses among isolated and grouped housed zebrafish^19^.

### Housing

L tanks were equipped with biological filters, under constant aeration with air stone and under 14-10 h light-dark regime. We covered all the tanks to prevent water evaporation and to avoid fish jumping out of the tanks. Barren tanks consist of tanks containing only water while enriched tanks received sand and gravel as a bottom substrate, caps for refuge and natural plants (two branches of *Cabombaceae* and *Pontederiaceae*). See the Fig. 2A.

Water temperature was maintained at 27 ± 1 °C; pH 7.0 ± 0.2; dissolved oxygen at 6.3 ± 0.3 mg/L; total ammonia at <0.01 mg/L; total hardness at 6 mg/L; and alkalinity at 22 mg/L CaCO_3_. We fed fish twice a day with TetraMin (Tetra, Melle, Germany). During the experimental period, we did not change the water or remove wastes.

### Stress Protocol

After the 15-day period, we applied an acute stress challenge in all fish by persecution with a pen net for 120 s. 15 minutes after the stress, fish were captured and immediately frozen in liquid nitrogen and stored at −20 °C until cortisol extraction. In order to prevent a possible stress response induced by manipulation, the time elapsed between capture and killing was less than 10 s. The 15-min time interval following stress is when cortisol level peaks^38^.

### Whole-body cortisol determination

The extraction and measurement of cortisol from zebrafish have been described in detail by Barcellos *et al*.^39^. Briefly, each fish was weighed and minced, and a pool of three fish were minced and placed into a disposable stomacher bag with phosphate buffered saline. The contents were transferred to test tube and ethyl ether was added. The tube was vortexed and centrifuged and then frozen at liquid nitrogen and the unfrozen portion (ethyl ether containing cortisol) was decanted. The ethyl ether was transferred to a new tube and completely evaporated under a gentle stream of nitrogen, yielding a lipid extract containing the cortisol. The extract was stored at −20 °C until the ELISA was conducted on the samples suspended with 1 ml of PBS buffer.

Whole-body cortisol was measured in duplicate samples of tissue extract with a commercially available enzyme-linked immunosorbent assay kit (EIAgen™ CORTISOL test, BioChem ImmunoSystems). The specificity of the test was evaluated by comparing the parallelism between the standard curve and serial dilutions of the tissue extracts in PBS. The standard curve constructed with the human standards ran in parallel to that obtained using serial dilutions of zebrafish tissue extracts. In the linear regression test, a high positive correlation was found between the curves. The intra-assay coefficient of variation was 3.33–3.65%

### Waterborne Concentrations of FLU and DZP

#### Sample Collection

To determine the concentrations of FLU and DZP in the water, 0.2-L samples were collected from tanks and stored in amber glass bottles on days 0, 3, 6, 10, and 15. For each analysis, triplicate water samples were analyzed. All samples were filtered using 0.22-μm filters before the extraction.

#### Solid Phase Extraction (SPE)

For FLU extraction, the SPE Strata-X cartridges were conditioned with 10 mL methanol followed by 10 mL HPLC-grade water. Water samples (50 mL) containing FLU were slowly passed through the SPE cartridges at a flow rate of approximately 10 mL/min. After extraction, the cartridges were kept wet, and serial washes with 10 mL of HPLC-grade water were performed before they were vacuum-dried for approximately 10 min. Samples were eluted with 2 mL methanol acidified with acetic acid 0.1% and collected in disposable glass tubes. The DZP was injected after filtering.

### LC-MS/MS Analysis

Analyses were performed using a Shimadzu LCMS-8040 triple quadrupole mass spectrometer (Japan) with a binary pump. The analytical column was an XR-ODS III (150 × 2 mm, 2.2 μm particle size). The mobile phase consisted of: (A) water with 0.1% formic acid and (B) methanol. For the gradient elution, the percentage of (B) changed linearly as follows: 0 min, 5%; 2.0 min, 30%; 4.0 min, 95%; and 5.0 min, 5%; for re-equilibration after each analysis. The flow rate used was 0.3 mL/min and the injection volume was 10 μL. Column temperature was set at 40 °C. The MS/MS analysis was performed using electrospray ionization (ESI), with the source in the positive-ion mode, and selected reaction monitoring (SRM) acquisition. The transition with the highest intensity was selected for quantification and the transition with the second highest intensity was used as confirmation. Quantification was performed using an external standard method with a ten-point calibration curve. Linearity was confirmed using the Anderson-Darling normality test^40^,^41^ homogeneity of variances using the Cochran’s test^42^, and independence of residues using the Durbin-Watson test^43^,^44^. Regression parameters were estimated by ordinary least squares.

### Statistical analysis

Cortisol data were analyzed by four-way ANOVA followed by Tukey’s multiple comparison test. FLU and DZP concentrations at 0 and 15 days after exposure were compared using a two-way ANOVA followed by the Tukey’s post hoc test. The homogeneity of variance was determined using Hartley’s test, and normality was assessed using the Kolmogorov–Smirnov test. Differences were considered significant at p < 0.05. Effect sizes were determined as partial Eta squared. The data are expressed as mean + standard error of mean (S.E.M).

## Additional Information

**How to cite this article**: Giacomini, A. C. V. V. *et al*. Environmental and Pharmacological Manipulations Blunt the Stress Response of Zebrafish in a Similar Manner. *Sci. Rep.* **6**, 28986; doi: 10.1038/srep28986 (2016).

## Supplementary Material

Supplementary Information

Supplementary Information

## References

[b1] Buzek J. & Chastel O. *Directive 2010/63/EU of the European Parliament and Council of the European Union.* Available at: http://eur-lex.europa.eu/LexUriServ/LexUriServ.do?uri=OJ:L:2010:276:0033:0079:en:PDF. (Accessed: 4^th^ September 2015) (2010).

[b2] Hajheidari S., Miladi-Gorji H. & Bigdeli I. Effects of environmental enrichment during induction of methamphetamine dependence on the behavioral withdrawal symptoms in rats. Neurosci Lett. 605, 39–43 (2015). 26275348 10.1016/j.neulet.2015.08.010

[b3] Goes T. C., Antunes F. D. & Teixeira-Silva F. Environmental enrichment for adult rats: effects on trait and state anxiety. Neurosci. Lett. 584, 93–96 (2015). 25316162 10.1016/j.neulet.2014.10.004

[b4] Kondo M. A. . Affective dysfunction in a mouse model of Rett syndrome: Therapeutic effects of environmental stimulation and physical activity. Dev Neurobiol. 76, 209–224 (2015). 26019053 10.1002/dneu.22308

[b5] Varman D. R. & Rajan K. E. Environmental Enrichment Reduces Anxiety by Differentially Activating Serotonergic and Neuropeptide Y (NPY)-Ergic System in Indian Field Mouse (*Mus booduga*): An Animal Model of Post-Traumatic Stress Disorder. PLoS One 10, e0127945 (2015). 26016844 10.1371/journal.pone.0127945 PMC4446351

[b6] Woo C. C., Donnelly J. H., Steinberg-Epstein R. & Leon M. Environmental enrichment as a therapy for autism: a clinical trial replication and extension. Behavioral Neuroscience 129, 412–422 (2015). 26052790 10.1037/bne0000068 PMC4682896

[b7] Manuel R. . The effects of environmental enrichment and age-related differences on inhibitory avoidance in zebrafish (*Danio rerio Hamilton*). Zebrafish 12, 152–165 (2015). 25646635 10.1089/zeb.2014.1045

[b8] Collymore C., Tolwani R. J. & Rasmussen S. The Behavioral Effects of Single Housing and Environmental Enrichment on Adult Zebrafish (Danio rerio). J. Am. Assoc. Lab. Anim. Sci. 54, 280–285 (2015). 26045453 PMC4460940

[b9] Howe K. . The zebrafish reference genome sequence and its relationship to the human genome. Nature 496, 498–505 (2013). 23594743 10.1038/nature12111 PMC3703927

[b10] Zon L. I. & Peterson R. T. *In vivo* drug discovery in the zebrafish. Nat. Rev. Drug Discov. 4, 35–44 (2005). 15688071 10.1038/nrd1606

[b11] Shin J. T. & Fishman M. C. From Zebrafish to human: modular medical models. Annu. Rev. Genomics Hum. Genet. 3, 311–340 (2002). 12142362 10.1146/annurev.genom.3.031402.131506

[b12] Goldsmith P. Zebrafish as a pharmacological tool: the how, why and when. Curr. Opin. Pharmacol. 4, 504–512 (2004). 15351356 10.1016/j.coph.2004.04.005

[b13] Abreu M. S. . Diazepam and fluoxetine decrease the stress response in zebrafish. PLoS ONE 9, e103232 (2014). 25054216 10.1371/journal.pone.0103232 PMC4108411

[b14] Idalencio R. . Waterborne Risperidone Decreases Stress Response in Zebrafish. PLoS ONE 10, e0140800 (2015). 26473477 10.1371/journal.pone.0140800 PMC4608780

[b15] Giacomini A. C. V. V. . Fluoxetine and diazepam acutely modulate stress induced-behavior. Behavioural Brain Research 296, 301–310 (2016). 26403161 10.1016/j.bbr.2015.09.027

[b16] Buske C. & Gerlai R. Shoaling develops with age in Zebrafish (*Danio rerio*). Prog. Neuropsychopharmacol Biol. Psych. 35, 1409–1415 (2011). 10.1016/j.pnpbp.2010.09.003 PMC3021101 20837077

[b17] Sneckser J. L., McRobert S. P., Murphy C. E. & Clotfelter E. D. Aggregation behavior in wild type and transgenic zebrafish. Ethology 112, 181–187 (2006).

[b18] Parker M. O., Millington M. E., Combe F. J. & Brennan C. H. Housing Conditions Differentially Affect Physiological and Behavioural Stress Responses of Zebrafish, as well as the Response to Anxiolytics. PLoS ONE 7, e34992 (2012). 22509375 10.1371/journal.pone.0034992 PMC3324417

[b19] Giacomini A. C. . My stress, our stress: Blunted cortisol response to stress in isolated housed zebrafish. Physiology & Behavior 139, 182–187 (2015). 25449397 10.1016/j.physbeh.2014.11.035

[b20] Mansur B. M., Santos B. R., Dias M. C. A. G., Pinheiro M. S. & Gouvea A. Effects of the number of subjects on the Dark/Ligth preference of zebrafish (Danio rerio). Zebrafish 11, 560–566 (2014). 25291497 10.1089/zeb.2014.0977

[b21] Brooks B. W. . Determinations of Select Antidepressant in Fish From an effuent-Dominated Stream. Environmental Toxicology and Chemistry 24, 464–469 (2005). 15720009 10.1897/04-081r.1

[b22] Gaworecki K. M. & Klaine S. J. Behavioral and biochemical responses of hybrid striped bass during and after fluoxetine exposure. Aquatic Toxicology 88, 207–213 (2008). 18547660 10.1016/j.aquatox.2008.04.011

[b23] Paterson G. & Metcalfe C. D. Uptake and depuration of the anti-depressant fluoxetine by the Japanese medaka (*Oryzias latipes*). Chemosphere 74, 125–130 (2008). 18845313 10.1016/j.chemosphere.2008.08.022

[b24] Sackerman J. . Zebrafish behavior in novel Enviroments, effects of acute exposure to anxiolytic compounds and choice of *Danio rerio* line. *International* Journal of Comparative Psychology 23, 43–61 (2010). PMC2879659 20523756

[b25] Calisto V., Domingues M. R. M. & Esteves V. I. Photodegradation of Psychiatric pharmaceuticals in aquatic environments – Kinetics and photodegradation products. Water Research 45, 6097–6106 (2011). 21943883 10.1016/j.watres.2011.09.008

[b26] Carvalho P. N., Basto M. C., Almeida C. M. R. & Brix H. A review of plant-pharmaceutical interactions: from uptake and effects in crop plants to phytoremediation in constructed wetlands. Environ Sci Pollut Res 21, 11729–11763 (2014). 10.1007/s11356-014-2550-3 24481515

[b27] Calisto V. & Esteves V. I. Psychiatric pharmaceuticals in the environment. Chemosphere 77, 1257–1274 (2009). 19815251 10.1016/j.chemosphere.2009.09.021

[b28] Jones O. A., Lester J. N. & Voulvoulis N. Pharmaceuticals: a threat to drinking water? TRENDS in Biotechnology 23, 163–167 (2005). 15780706 10.1016/j.tibtech.2005.02.001

[b29] Ternes T., Bonerz M. & Schmidt T. Determination of neutral pharmaceuticals in wastewater and rivers by liquid chromatography–electrospray tandem mass spectrometry. J. Chromatogr. 938, 175–185 (2001). 10.1016/s0021-9673(01)01205-5 11771837

[b30] Kolpin D. W. . Pharmaceuticals, hormones, and other organic wastewater contaminants in US streams, 1999-2000: a national reconnaissance. Environmental Science and Technology 36, 1202–1211 (2002). 11944670 10.1021/es011055j

[b31] Abreu M. S. . Effects of waterborne fluoxetine on stress response and osmoregulation in zebrafish. Environmental Toxicology and Pharmacology 40, 704–707 (2015). 26414864 10.1016/j.etap.2015.09.001

[b32] Alzaid A. . Cold-induced changes in stress hormone and steroidogenic transcript levels in cunner (Tautogolabrus adspersus), a fish capable of metabolic depression. Gen Comp Endocrinol. 224, 126–135 (2015). 26188716 10.1016/j.ygcen.2015.07.007

[b33] Pham T. M. . Housing environment influences the need for pain relief during post-operative recovery in mice. Physiology & Behavior 99, 663–668 (2010). 20149809 10.1016/j.physbeh.2010.01.038

[b34] Vachon P. . Alleviation of chronic neuropathic pain by environmental enrichment in mice well after the establishment of chronic pain. Behav. Brain Funct. 9, 1–9 (2013). 24025218 10.1186/1744-9081-9-22 PMC3679946

[b35] Peck J. A. . Environmental enrichment induces early heroin abstinence in an animal conflict model. Pharmacology, Biochemistry and Behavior 138, 20–25 (2015). 10.1016/j.pbb.2015.09.009 26368843

[b36] Ahmadalipour . Effects of environmental enrichment on behavioral deficits and alterations in hippocampal BDNF induced by prenatal exposure to morphine in juvenile rats. Neuroscience 305, 372–383 (2015). 26272536 10.1016/j.neuroscience.2015.08.015

[b37] Favre M. R. . Predictable enriched environment prevents development of hyper-emotionality in the VPA rat model of autism. Frontiers in Neuroscience 9, 1–14 (2015). 26089770 10.3389/fnins.2015.00127 PMC4452729

[b38] Ramsay J. M. . Whole body cortisol response of zebrafish to acute net handling stress. Aquaculture 297, 157–162 (2009). 25587201 10.1016/j.aquaculture.2009.08.035 PMC4289633

[b39] Barcellos L. J. G. . Whole-body cortisol increases after direct and visual contact with the predator in zebrafish. Danio rerio. Aquaculture 272, 774–778 (2007).

[b40] Razali N. M. & Wah Y. B. Power comparisons of Shapiro-Wilk, Kolmogorov-Smirnov, Lillefors and Anderson-Darling tests. Journal of Multivariate Analysis 2, 23–33 (2011).

[b41] Pettitt A. N. Testing the normality of Several Independent Samples using the Anderson-Darling Statistic. Journal of the Royal Statistical Society 26, 156–161 (1977).

[b42] AOAC International, *Appendix D: Guidelines for Collaborative Study Procedures To Validate Characteristics of a Method of Analysis.* Available at: http://www.eoma.aoac.org/app_d.pdf. (Accessed: 10^th^ August 2015) (2005).

[b43] Ali M. M. & Sharma S. C. Robustness to non normality of the Durbin-Watson test for autocorrelation. Journal of Econometrics 57, 117–136 (1993).

[b44] Souza S. V. C. & Junqueira R. G. A procedure to assess linearity by ordinary least Squares Method. Analytica Chimica Acta 552, 25–35 (2005).

