# Supplementary material for: Environmental and Pharmacological Manipulations Blunt the Stress Response of Zebrafish in a Similar Manner
Source: Sci Rep. 2016 Jun 28;6:28986. doi: 10.1038/srep28986 (PMC4924565; doi:10.1038/srep28986)
Supplement: Supplementary Information [file srep28986-s1.pdf]

```
UNIANOVA Cortisol BY Housing Environment Stress Drug
  /METHOD=SSTYPE(3)
  /INTERCEPT=INCLUDE
  /EMMEANS=TABLES(OVERALL)
  /PRINT=ETASQ DESCRIPTIVE
  /CRITERIA=ALPHA(.05)
  /DESIGN=Housing Environment Stress Drug Housing*Environment Housing*Stre
ss Housing*Drug Environment*Stress Environment*Drug Stress*Drug Housing*En
vironment*Stress Housing*Environment*Drug Housing*Stress*Drug Environment*
Stress*Drug
Housing*Environment*Stress*Drug.
```

Univariate Analysis of Variance

[DataSet5] C:\Users\Angelo\Dropbox\UFRGS HOME\ORIENTAÇÕES\Ana Giacominni-UPF\Enriquecimento\DZP data.sav

Between-Subjects Factors

		Value Label	N
Housing	GRUPO	GRUPO	72
	ISOLADO	ISOLADO	44
Environment	EA-	EA-	63
	EA+	EA+	53
Stress	S-	S-	53
	S+	S+	63
Drug	1	Dzp0	53
	Dzp16	Dzp16	63

Descriptive Statistics

Dependent Variable: Cortisol

Housing	Environment	Stress	Drug	Mean	Std. Deviation	N
GRUPO	EA-	S-	Dzp0	1,6000	,65192	5
			Dzp16	2,8182	2,26134	11
			Total	2,4375	1,96532	16
		S+	Dzp0	58,8889	15,82280	9
			Dzp16	17,4545	6,38535	11
			Total	36,1000	23,96137	20
		Total	Dzp0	38,4286	31,07550	14
			Dzp16	10,1364	8,82931	22
			Total	21,1389	24,51781	36
	EA+	S-	Dzp0	2,3333	,93095	6
			Dzp16	1,9000	,69921	10
			Total	2,0625	,79320	16
		S+	Dzp0	13,2273	7,97923	11
			Dzp16	6,1944	3,18634	9
			Total	10,0625	7,11830	20
		Total	Dzp0	9,3824	8,29821	17
			Dzp16	3,9342	3,10000	19
			Total	6,5069	6,63553	36
	Total	S-	Dzp0	2,0000	,86603	11
			Dzp16	2,3810	1,73136	21
			Total	2,2500	1,48650	32
		S+	Dzp0	33,7750	26,11738	20
			Dzp16	12,3875	7,66592	20
			Total	23,0813	21,86852	40
		Total	Dzp0	22,5000	25,90560	31
			Dzp16	7,2622	7,41998	41
			Total	13,8229	19,29535	72
ISOLADO	EA-	S-	Dzp0	4,7957	,36395	7
			Dzp16	1,4167	,49160	6
			Total	3,2362	1,80028	13
		S+	Dzp0	11,9167	2,31120	6
			Dzp16	22,5000	10,33717	8
			Total	17,9643	9,44104	14
		Total	Dzp0	8,0823	3,99300	13
			Dzp16	13,4643	13,22362	14
			Total	10,8730	10,11437	27
	EA+	S-	Dzp0	2,3750	,85391	4
			Dzp16	2,1250	,94648	4
			Total	2,2500	,84515	8

Descriptive Statistics

Dependent Variable: Cortisol

Housing	Environment	Stress	Drug	Mean	Std. Deviation	N
		S+	Dzp0	10,2000	3,91472	5
			Dzp16	7,4375	4,46456	4
			Total	8,9722	4,15415	9
		Total	Dzp0	6,7222	4,99444	9
			Dzp16	4,7813	4,12189	8
			Total	5,8088	4,57193	17
	Total	S-	Dzp0	3,9155	1,33784	11
			Dzp16	1,7000	,75277	10
			Total	2,8605	1,56058	21
		S+	Dzp0	11,1364	3,09912	11
			Dzp16	17,4792	11,33301	12
			Total	14,4457	8,89265	23
		Total	Dzp0	7,5259	4,36830	22
			Dzp16	10,3068	11,49739	22
			Total	8,9164	8,70950	44
Total	EA-	S-	Dzp0	3,4642	1,71309	12
			Dzp16	2,3235	1,93602	17
			Total	2,7955	1,90305	29
		S+	Dzp0	40,1000	26,68948	15
			Dzp16	19,5789	8,41174	19
			Total	28,6324	21,16039	34
		Total	Dzp0	23,8174	26,99913	27
			Dzp16	11,4306	10,69723	36
			Total	16,7392	20,21063	63
	EA+	S-	Dzp0	2,3500	,85147	10
			Dzp16	1,9643	,74587	14
			Total	2,1250	,79741	24
		S+	Dzp0	12,2812	6,97369	16
			Dzp16	6,5769	3,47968	13
			Total	9,7241	6,29106	29
		Total	Dzp0	8,4615	7,32929	26
			Dzp16	4,1852	3,37382	27
			Total	6,2830	6,01461	53
	Total	S-	Dzp0	2,9577	1,47321	22
			Dzp16	2,1613	1,50769	31
			Total	2,4919	1,53130	53
		S+	Dzp0	25,7419	23,58915	31
			Dzp16	14,2969	9,37362	32
			Total	19,9286	18,61313	63

Descriptive Statistics

Dependent Variable: Cortisol

Housing	Environment	Stress	Drug	Mean	Std. Deviation	N
		Total	Dzp0	16,2843	21,22182	53
			Dzp16	8,3254	9,07936	63
			Total	11,9618	16,24629	116

Tests of Between-Subjects Effects

Dependent Variable: Cortisol

Source	Type III Sum of Squares	df	Mean Square	F	Sig.
Corrected Model	26260,540[a]	15	1750,703	42,775	,000
Intercept	11123,111	1	11123,111	271,774	,000
Housing	690,357	1	690,357	16,868	,000
Environment	2274,379	1	2274,379	55,571	,000
Stress	6566,679	1	6566,679	160,446	,000
Drug	752,715	1	752,715	18,391	,000
Housing * Environment	593,408	1	593,408	14,499	,000
Housing * Stress	833,754	1	833,754	20,371	,000
Housing * Drug	1070,884	1	1070,884	26,165	,000
Environment * Stress	2051,795	1	2051,795	50,132	,000
Environment * Drug	202,063	1	202,063	4,937	,029
Stress * Drug	568,688	1	568,688	13,895	,000
Housing * Environment * Stress	691,018	1	691,018	16,884	,000
Housing * Environment * Drug	734,694	1	734,694	17,951	,000
Housing * Stress * Drug	1466,377	1	1466,377	35,828	,000
Environment * Stress * Drug	152,540	1	152,540	3,727	,056
Housing * Environment * Stress * Drug	1098,048	1	1098,048	26,829	,000
Error	4092,773	100	40,928		
Total	46951,162	116			
Corrected Total	30353,313	115			

Tests of Between-Subjects Effects

Dependent Variable: Cortisol

Source	Partial Eta Squared
Corrected Model	,865
Intercept	,731
Housing	,144
Environment	,357
Stress	,616
Drug	,155
Housing * Environment	,127
Housing * Stress	,169
Housing * Drug	,207
Environment * Stress	,334
Environment * Drug	,047
Stress * Drug	,122
Housing * Environment * Stress	,144
Housing * Environment * Drug	,152
Housing * Stress * Drug	,264
Environment * Stress * Drug	,036
Housing * Environment * Stress * Drug	,212
Error	
Total	
Corrected Total	

a. R Squared = ,865 (Adjusted R Squared = ,845)

Estimated Marginal Means

Grand Mean

Dependent Variable: Cortisol

Mean	Std. Error	95% Confidence Interval	
		Lower Bound	Upper Bound
10,449	,634	9,191	11,706

```
UNIANOVA Cortisol BY Grupo
  /METHOD=SSTYPE(3)
  /INTERCEPT=INCLUDE
  /POSTHOC=Grupo(TUKEY)
  /EMMEANS=TABLES(OVERALL)
  /PRINT=ETASQ DESCRIPTIVE
  /CRITERIA=ALPHA(.05)
  /DESIGN=Grupo.
```

Univariate Analysis of Variance

[DataSet5] C:\Users\Angelo\Dropbox\UFRGS HOME\ORIENTAÇÕES\Ana Giacominni-UPF\Enriquecimento\DZP data.sav

Between-Subjects Factors

		Value Label	N
Grupo	GEA-S-cr	GEA-S-cr	5
	GEA-S-D	GEA-S-D	11
	GEA-S+cr	GEA-S+cr	9
	GEA-S+D	GEA-S+D	11
	GEA+S-cr	GEA+S-cr	6
	GEA+S-D	GEA+S-D	10
	GEA+S+cr	GEA+S+cr	11
	GEA+S+D	GEA+S+D	9
	IEA-S-cr	IEA-S-cr	7
	IEA-S-D	IEA-S-D	6
	IEA-S+cr	IEA-S+cr	6
	IEA-S+D	IEA-S+D	8
	IEA+S-cr	IEA+S-cr	4
	IEA+S-D	IEA+S-D	4
	IEA+S+cr	IEA+S+cr	5
	IEA+S+D	IEA+S+D	4

Descriptive Statistics

Dependent Variable: Cortisol

Grupo	Mean	Std. Deviation	N
GEA-S-cr	1,6000	,65192	5
GEA-S-D	2,8182	2,26134	11
GEA-S+cr	58,8889	15,82280	9
GEA-S+D	17,4545	6,38535	11
GEA+S-cr	2,3333	,93095	6
GEA+S-D	1,9000	,69921	10
GEA+S+cr	13,2273	7,97923	11
GEA+S+D	6,1944	3,18634	9
IEA-S-cr	4,7957	,36395	7
IEA-S-D	1,4167	,49160	6
IEA-S+cr	11,9167	2,31120	6
IEA-S+D	22,5000	10,33717	8
IEA+S-cr	2,3750	,85391	4
IEA+S-D	2,1250	,94648	4
IEA+S+cr	10,2000	3,91472	5
IEA+S+D	7,4375	4,46456	4
Total	11,9618	16,24629	116

Tests of Between-Subjects Effects

Dependent Variable: Cortisol

Source	Type III Sum of Squares	df	Mean Square	F	Sig.	Partial Eta Squared
Corrected Model	26260,540[a]	15	1750,703	42,775	,000	,865
Intercept	11123,111	1	11123,111	271,774	,000	,731
Grupo	26260,540	15	1750,703	42,775	,000	,865
Error	4092,773	100	40,928			
Total	46951,162	116				
Corrected Total	30353,313	115				

a. R Squared = ,865 (Adjusted R Squared = ,845)

Estimated Marginal Means

Grand Mean

Dependent Variable: Cortisol

Mean	Std. Error	95% Confidence Interval	
		Lower Bound	Upper Bound
10,449	,634	9,191	11,706

Post Hoc Tests

Grupo

Multiple Comparisons

Dependent Variable: Cortisol
Tukey HSD

(I) Grupo	(J) Grupo	Mean Difference (I-J)	Std. Error	Sig.	95% Confidence Interval	
					Lower Bound	Upper Bound
GEA-S-cr	GEA-S-D	-1,2182	3,45054	1,000	-13,3467	10,9104
	GEA-S+cr	-57,2889*	3,56834	,000	-69,8315	-44,7463
	GEA-S+D	-15,8545*	3,45054	,001	-27,9831	-3,7260
	GEA+S-cr	-,7333	3,87387	1,000	-14,3498	12,8832
	GEA+S-D	-,3000	3,50404	1,000	-12,6166	12,0166
	GEA+S+cr	-11,6273	3,45054	,075	-23,7558	,5013
	GEA+S+D	-4,5944	3,56834	,996	-17,1371	7,9482
	IEA-S-cr	-3,1957	3,74598	1,000	-16,3627	9,9713
	IEA-S-D	,1833	3,87387	1,000	-13,4332	13,7998
	IEA-S+cr	-10,3167	3,87387	,369	-23,9332	3,2998
	IEA-S+D	-20,9000*	3,64712	,000	-33,7195	-8,0805
	IEA+S-cr	-,7750	4,29156	1,000	-15,8597	14,3097
	IEA+S-D	-,5250	4,29156	1,000	-15,6097	14,5597
	IEA+S+cr	-8,6000	4,04612	,744	-22,8220	5,6220
	IEA+S+D	-5,8375	4,29156	,992	-20,9222	9,2472
GEA-S-D	GEA-S-cr	1,2182	3,45054	1,000	-10,9104	13,3467
	GEA-S+cr	-56,0707*	2,87545	,000	-66,1778	-45,9636
	GEA-S+D	-14,6364*	2,72789	,000	-24,2248	-5,0479
	GEA+S-cr	,4848	3,24684	1,000	-10,9277	11,8974
	GEA+S-D	,9182	2,79526	1,000	-8,9071	10,7434
	GEA+S+cr	-10,4091*	2,72789	,020	-19,9975	-,8206
	GEA+S+D	-3,3763	2,87545	,998	-13,4834	6,7309
	IEA-S-cr	-1,9775	3,09314	1,000	-12,8498	8,8948
	IEA-S-D	1,4015	3,24684	1,000	-10,0110	12,8141
	IEA-S+cr	-9,0985	3,24684	,285	-20,5110	2,3141
	IEA-S+D	-19,6818*	2,97265	,000	-30,1306	-9,2330
	IEA+S-cr	,4432	3,73532	1,000	-12,6864	13,5727
	IEA+S-D	,6932	3,73532	1,000	-12,4364	13,8227
	IEA+S+cr	-7,3818	3,45054	,735	-19,5104	4,7467
	IEA+S+D	-4,6193	3,73532	,997	-17,7489	8,5102
GEA-S+cr	GEA-S-cr	57,2889*	3,56834	,000	44,7463	69,8315
	GEA-S-D	56,0707*	2,87545	,000	45,9636	66,1778
	GEA-S+D	41,4343*	2,87545	,000	31,3272	51,5415
	GEA+S-cr	56,5556*	3,37177	,000	44,7039	68,4072
	GEA+S-D	56,9889*	2,93944	,000	46,6569	67,3209
	GEA+S+cr	45,6616*	2,87545	,000	35,5545	55,7687
	GEA+S+D	52,6944*	3,01580	,000	42,0940	63,2949
	IEA-S-cr	54,0932*	3,22403	,000	42,7608	65,4255
	IEA-S-D	57,4722*	3,37177	,000	45,6206	69,3239

Multiple Comparisons

Dependent Variable: Cortisol
Tukey HSD

(I) Grupo	(J) Grupo	Mean Difference (I-J)	Std. Error	Sig.	95% Confidence Interval	
					Lower Bound	Upper Bound
	IEA-S+cr	46,9722*	3,37177	,000	35,1206	58,8239
	IEA-S+D	36,3889*	3,10862	,000	25,4622	47,3156
	IEA+S-cr	56,5139*	3,84441	,000	43,0009	70,0269
	IEA+S-D	56,7639*	3,84441	,000	43,2509	70,2769
	IEA+S+cr	48,6889*	3,56834	,000	36,1463	61,2315
	IEA+S+D	51,4514*	3,84441	,000	37,9384	64,9644
GEA-S+D	GEA-S-cr	15,8545*	3,45054	,001	3,7260	27,9831
	GEA-S-D	14,6364*	2,72789	,000	5,0479	24,2248
	GEA-S+cr	-41,4343*	2,87545	,000	-51,5415	-31,3272
	GEA+S-cr	15,1212*	3,24684	,001	3,7087	26,5338
	GEA+S-D	15,5545*	2,79526	,000	5,7293	25,3798
	GEA+S+cr	4,2273	2,72789	,973	-5,3612	13,8157
	GEA+S+D	11,2601*	2,87545	,015	1,1530	21,3672
	IEA-S-cr	12,6588*	3,09314	,008	1,7865	23,5311
	IEA-S-D	16,0379*	3,24684	,000	4,6253	27,4504
	IEA-S+cr	5,5379	3,24684	,941	-5,8747	16,9504
	IEA-S+D	-5,0455	2,97265	,943	-15,4942	5,4033
	IEA+S-cr	15,0795*	3,73532	,010	1,9500	28,2091
	IEA+S-D	15,3295*	3,73532	,008	2,2000	28,4591
	IEA+S+cr	7,2545	3,45054	,759	-4,8740	19,3831
	IEA+S+D	10,0170	3,73532	,357	-3,1125	23,1466
GEA+S-cr	GEA-S-cr	,7333	3,87387	1,000	-12,8832	14,3498
	GEA-S-D	-,4848	3,24684	1,000	-11,8974	10,9277
	GEA-S+cr	-56,5556*	3,37177	,000	-68,4072	-44,7039
	GEA-S+D	-15,1212*	3,24684	,001	-26,5338	-3,7087
	GEA+S-D	,4333	3,30364	1,000	-11,1789	12,0455
	GEA+S+cr	-10,8939	3,24684	,078	-22,3065	,5186
	GEA+S+D	-3,8611	3,37177	,999	-15,7128	7,9905
	IEA-S-cr	-2,4624	3,55923	1,000	-14,9730	10,0482
	IEA-S-D	,9167	3,69359	1,000	-12,0662	13,8995
	IEA-S+cr	-9,5833	3,69359	,414	-22,5662	3,3995
	IEA-S+D	-20,1667*	3,45503	,000	-32,3110	-8,0223
	IEA+S-cr	-,0417	4,12955	1,000	-14,5569	14,4736
	IEA+S-D	,2083	4,12955	1,000	-14,3069	14,7236
	IEA+S+cr	-7,8667	3,87387	,802	-21,4832	5,7498
	IEA+S+D	-5,1042	4,12955	,997	-19,6194	9,4111
GEA+S-D	GEA-S-cr	,3000	3,50404	1,000	-12,0166	12,6166
	GEA-S-D	-,9182	2,79526	1,000	-10,7434	8,9071
	GEA-S+cr	-56,9889*	2,93944	,000	-67,3209	-46,6569

Multiple Comparisons

Dependent Variable: Cortisol
Tukey HSD

(I) Grupo	(J) Grupo	Mean Difference (I-J)	Std. Error	Sig.	95% Confidence Interval	
					Lower Bound	Upper Bound
	GEA-S+D	-15,5545*	2,79526	,000	-25,3798	-5,7293
	GEA+S-cr	-,4333	3,30364	1,000	-12,0455	11,1789
	GEA+S+cr	-11,3273*	2,79526	,009	-21,1525	-1,5020
	GEA+S+D	-4,2944	2,93944	,984	-14,6265	6,0376
	IEA-S-cr	-2,8957	3,15271	1,000	-13,9774	8,1860
	IEA-S-D	,4833	3,30364	1,000	-11,1289	12,0955
	IEA-S+cr	-10,0167	3,30364	,175	-21,6289	1,5955
	IEA-S+D	-20,6000*	3,03459	,000	-31,2665	-9,9335
	IEA+S-cr	-,4750	3,78480	1,000	-13,7784	12,8284
	IEA+S-D	-,2250	3,78480	1,000	-13,5284	13,0784
	IEA+S+cr	-8,3000	3,50404	,575	-20,6166	4,0166
	IEA+S+D	-5,5375	3,78480	,984	-18,8409	7,7659
GEA+S+cr	GEA-S-cr	11,6273	3,45054	,075	-,5013	23,7558
	GEA-S-D	10,4091*	2,72789	,020	,8206	19,9975
	GEA-S+cr	-45,6616*	2,87545	,000	-55,7687	-35,5545
	GEA-S+D	-4,2273	2,72789	,973	-13,8157	5,3612
	GEA+S-cr	10,8939	3,24684	,078	-,5186	22,3065
	GEA+S-D	11,3273*	2,79526	,009	1,5020	21,1525
	GEA+S+D	7,0328	2,87545	,519	-3,0743	17,1400
	IEA-S-cr	8,4316	3,09314	,330	-2,4407	19,3038
	IEA-S-D	11,8106*	3,24684	,035	,3981	23,2232
	IEA-S+cr	1,3106	3,24684	1,000	-10,1019	12,7232
	IEA-S+D	-9,2727	2,97265	,143	-19,7215	1,1761
	IEA+S-cr	10,8523	3,73532	,231	-2,2773	23,9818
	IEA+S-D	11,1023	3,73532	,200	-2,0273	24,2318
	IEA+S+cr	3,0273	3,45054	1,000	-9,1013	15,1558
	IEA+S+D	5,7898	3,73532	,973	-7,3398	18,9193
GEA+S+D	GEA-S-cr	4,5944	3,56834	,996	-7,9482	17,1371
	GEA-S-D	3,3763	2,87545	,998	-6,7309	13,4834
	GEA-S+cr	-52,6944*	3,01580	,000	-63,2949	-42,0940
	GEA-S+D	-11,2601*	2,87545	,015	-21,3672	-1,1530
	GEA+S-cr	3,8611	3,37177	,999	-7,9905	15,7128
	GEA+S-D	4,2944	2,93944	,984	-6,0376	14,6265
	GEA+S+cr	-7,0328	2,87545	,519	-17,1400	3,0743
	IEA-S-cr	1,3987	3,22403	1,000	-9,9336	12,7311
	IEA-S-D	4,7778	3,37177	,988	-7,0739	16,6294
	IEA-S+cr	-5,7222	3,37177	,943	-17,5739	6,1294
	IEA-S+D	-16,3056*	3,10862	,000	-27,2322	-5,3789
	IEA+S-cr	3,8194	3,84441	1,000	-9,6935	17,3324

Multiple Comparisons

Dependent Variable: Cortisol
Tukey HSD

(I) Grupo	(J) Grupo	Mean Difference (I-J)	Std. Error	Sig.	95% Confidence Interval Lower Bound	Upper Bound
	IEA+S-D	4,0694	3,84441	,999	-9,4435	17,5824
	IEA+S+cr	-4,0056	3,56834	,999	-16,5482	8,5371
	IEA+S+D	-1,2431	3,84441	1,000	-14,7560	12,2699
IEA-S-cr	GEA-S-cr	3,1957	3,74598	1,000	-9,9713	16,3627
	GEA-S-D	1,9775	3,09314	1,000	-8,8948	12,8498
	GEA-S+cr	-54,0932*	3,22403	,000	-65,4255	-42,7608
	GEA-S+D	-12,6588*	3,09314	,008	-23,5311	-1,7865
	GEA+S-cr	2,4624	3,55923	1,000	-10,0482	14,9730
	GEA+S-D	2,8957	3,15271	1,000	-8,1860	13,9774
	GEA+S+cr	-8,4316	3,09314	,330	-19,3038	2,4407
	GEA+S+D	-1,3987	3,22403	1,000	-12,7311	9,9336
	IEA-S-D	3,3790	3,55923	1,000	-9,1315	15,8896
	IEA-S+cr	-7,1210	3,55923	,819	-19,6315	5,3896
	IEA-S+D	-17,7043*	3,31101	,000	-29,3424	-6,0662
	IEA+S-cr	2,4207	4,00983	1,000	-11,6737	16,5151
	IEA+S-D	2,6707	4,00983	1,000	-11,4237	16,7651
	IEA+S+cr	-5,4043	3,74598	,986	-18,5713	7,7627
	IEA+S+D	-2,6418	4,00983	1,000	-16,7362	11,4526
IEA-S-D	GEA-S-cr	-,1833	3,87387	1,000	-13,7998	13,4332
	GEA-S-D	-1,4015	3,24684	1,000	-12,8141	10,0110
	GEA-S+cr	-57,4722*	3,37177	,000	-69,3239	-45,6206
	GEA-S+D	-16,0379*	3,24684	,000	-27,4504	-4,6253
	GEA+S-cr	-,9167	3,69359	1,000	-13,8995	12,0662
	GEA+S-D	-,4833	3,30364	1,000	-12,0955	11,1289
	GEA+S+cr	-11,8106*	3,24684	,035	-23,2232	-,3981
	GEA+S+D	-4,7778	3,37177	,988	-16,6294	7,0739
	IEA-S-cr	-3,3790	3,55923	1,000	-15,8896	9,1315
	IEA-S+cr	-10,5000	3,69359	,263	-23,4828	2,4828
	IEA-S+D	-21,0833*	3,45503	,000	-33,2277	-8,9390
	IEA+S-cr	-,9583	4,12955	1,000	-15,4736	13,5569
	IEA+S-D	-,7083	4,12955	1,000	-15,2236	13,8069
	IEA+S+cr	-8,7833	3,87387	,648	-22,3998	4,8332
	IEA+S+D	-6,0208	4,12955	,985	-20,5361	8,4944
IEA-S+cr	GEA-S-cr	10,3167	3,87387	,369	-3,2998	23,9332
	GEA-S-D	9,0985	3,24684	,285	-2,3141	20,5110
	GEA-S+cr	-46,9722*	3,37177	,000	-58,8239	-35,1206
	GEA-S+D	-5,5379	3,24684	,941	-16,9504	5,8747
	GEA+S-cr	9,5833	3,69359	,414	-3,3995	22,5662
	GEA+S-D	10,0167	3,30364	,175	-1,5955	21,6289

Multiple Comparisons

Dependent Variable: Cortisol
Tukey HSD

(I) Grupo	(J) Grupo	Mean Difference (I-J)	Std. Error	Sig.	95% Confidence Interval	
					Lower Bound	Upper Bound
	GEA+S+cr	-1,3106	3,24684	1,000	-12,7232	10,1019
	GEA+S+D	5,7222	3,37177	,943	-6,1294	17,5739
	IEA-S-cr	7,1210	3,55923	,819	-5,3896	19,6315
	IEA-S-D	10,5000	3,69359	,263	-2,4828	23,4828
	IEA-S+D	-10,5833	3,45503	,163	-22,7277	1,5610
	IEA+S-cr	9,5417	4,12955	,617	-4,9736	24,0569
	IEA+S-D	9,7917	4,12955	,573	-4,7236	24,3069
	IEA+S+cr	1,7167	3,87387	1,000	-11,8998	15,3332
	IEA+S+D	4,4792	4,12955	,999	-10,0361	18,9944
IEA-S+D	GEA-S-cr	20,9000*	3,64712	,000	8,0805	33,7195
	GEA-S-D	19,6818*	2,97265	,000	9,2330	30,1306
	GEA-S+cr	-36,3889*	3,10862	,000	-47,3156	-25,4622
	GEA-S+D	5,0455	2,97265	,943	-5,4033	15,4942
	GEA+S-cr	20,1667*	3,45503	,000	8,0223	32,3110
	GEA+S-D	20,6000*	3,03459	,000	9,9335	31,2665
	GEA+S+cr	9,2727	2,97265	,143	-1,1761	19,7215
	GEA+S+D	16,3056*	3,10862	,000	5,3789	27,2322
	IEA-S-cr	17,7043*	3,31101	,000	6,0662	29,3424
	IEA-S-D	21,0833*	3,45503	,000	8,9390	33,2277
	IEA-S+cr	10,5833	3,45503	,163	-1,5610	22,7277
	IEA+S-cr	20,1250*	3,91764	,000	6,3546	33,8954
	IEA+S-D	20,3750*	3,91764	,000	6,6046	34,1454
	IEA+S+cr	12,3000	3,64712	,074	-,5195	25,1195
	IEA+S+D	15,0625*	3,91764	,018	1,2921	28,8329
IEA+S-cr	GEA-S-cr	,7750	4,29156	1,000	-14,3097	15,8597
	GEA-S-D	-,4432	3,73532	1,000	-13,5727	12,6864
	GEA-S+cr	-56,5139*	3,84441	,000	-70,0269	-43,0009
	GEA-S+D	-15,0795*	3,73532	,010	-28,2091	-1,9500
	GEA+S-cr	,0417	4,12955	1,000	-14,4736	14,5569
	GEA+S-D	,4750	3,78480	1,000	-12,8284	13,7784
	GEA+S+cr	-10,8523	3,73532	,231	-23,9818	2,2773
	GEA+S+D	-3,8194	3,84441	1,000	-17,3324	9,6935
	IEA-S-cr	-2,4207	4,00983	1,000	-16,5151	11,6737
	IEA-S-D	,9583	4,12955	1,000	-13,5569	15,4736
	IEA-S+cr	-9,5417	4,12955	,617	-24,0569	4,9736
	IEA-S+D	-20,1250*	3,91764	,000	-33,8954	-6,3546
	IEA+S-D	,2500	4,52370	1,000	-15,6507	16,1507
	IEA+S+cr	-7,8250	4,29156	,902	-22,9097	7,2597
	IEA+S+D	-5,0625	4,52370	,999	-20,9632	10,8382

Multiple Comparisons

Dependent Variable: Cortisol
Tukey HSD

(I) Grupo	(J) Grupo	Mean Difference (I-J)	Std. Error	Sig.	95% Confidence Interval	
					Lower Bound	Upper Bound
IEA+S-D	GEA-S-cr	,5250	4,29156	1,000	-14,5597	15,6097
	GEA-S-D	-,6932	3,73532	1,000	-13,8227	12,4364
	GEA-S+cr	-56,7639*	3,84441	,000	-70,2769	-43,2509
	GEA-S+D	-15,3295*	3,73532	,008	-28,4591	-2,2000
	GEA+S-cr	-,2083	4,12955	1,000	-14,7236	14,3069
	GEA+S-D	,2250	3,78480	1,000	-13,0784	13,5284
	GEA+S+cr	-11,1023	3,73532	,200	-24,2318	2,0273
	GEA+S+D	-4,0694	3,84441	,999	-17,5824	9,4435
	IEA-S-cr	-2,6707	4,00983	1,000	-16,7651	11,4237
	IEA-S-D	,7083	4,12955	1,000	-13,8069	15,2236
	IEA-S+cr	-9,7917	4,12955	,573	-24,3069	4,7236
	IEA-S+D	-20,3750*	3,91764	,000	-34,1454	-6,6046
	IEA+S-cr	-,2500	4,52370	1,000	-16,1507	15,6507
	IEA+S+cr	-8,0750	4,29156	,878	-23,1597	7,0097
	IEA+S+D	-5,3125	4,52370	,998	-21,2132	10,5882
IEA+S+cr	GEA-S-cr	8,6000	4,04612	,744	-5,6220	22,8220
	GEA-S-D	7,3818	3,45054	,735	-4,7467	19,5104
	GEA-S+cr	-48,6889*	3,56834	,000	-61,2315	-36,1463
	GEA-S+D	-7,2545	3,45054	,759	-19,3831	4,8740
	GEA+S-cr	7,8667	3,87387	,802	-5,7498	21,4832
	GEA+S-D	8,3000	3,50404	,575	-4,0166	20,6166
	GEA+S+cr	-3,0273	3,45054	1,000	-15,1558	9,1013
	GEA+S+D	4,0056	3,56834	,999	-8,5371	16,5482
	IEA-S-cr	5,4043	3,74598	,986	-7,7627	18,5713
	IEA-S-D	8,7833	3,87387	,648	-4,8332	22,3998
	IEA-S+cr	-1,7167	3,87387	1,000	-15,3332	11,8998
	IEA-S+D	-12,3000	3,64712	,074	-25,1195	,5195
	IEA+S-cr	7,8250	4,29156	,902	-7,2597	22,9097
	IEA+S-D	8,0750	4,29156	,878	-7,0097	23,1597
	IEA+S+D	2,7625	4,29156	1,000	-12,3222	17,8472
IEA+S+D	GEA-S-cr	5,8375	4,29156	,992	-9,2472	20,9222
	GEA-S-D	4,6193	3,73532	,997	-8,5102	17,7489
	GEA-S+cr	-51,4514*	3,84441	,000	-64,9644	-37,9384
	GEA-S+D	-10,0170	3,73532	,357	-23,1466	3,1125
	GEA+S-cr	5,1042	4,12955	,997	-9,4111	19,6194
	GEA+S-D	5,5375	3,78480	,984	-7,7659	18,8409
	GEA+S+cr	-5,7898	3,73532	,973	-18,9193	7,3398
	GEA+S+D	1,2431	3,84441	1,000	-12,2699	14,7560
	IEA-S-cr	2,6418	4,00983	1,000	-11,4526	16,7362

Multiple Comparisons

Dependent Variable: Cortisol
Tukey HSD

(I) Grupo	(J) Grupo	Mean Difference (I-J)	Std. Error	Sig.	95% Confidence Interval Lower Bound	Upper Bound
	IEA-S-D	6,0208	4,12955	,985	-8,4944	20,5361
	IEA-S+cr	-4,4792	4,12955	,999	-18,9944	10,0361
	IEA-S+D	-15,0625*	3,91764	,018	-28,8329	-1,2921
	IEA+S-cr	5,0625	4,52370	,999	-10,8382	20,9632
	IEA+S-D	5,3125	4,52370	,998	-10,5882	21,2132
	IEA+S+cr	-2,7625	4,29156	1,000	-17,8472	12,3222

Dependent Variable: Cortisol
Tukey HSD

Homogeneous Subsets

Cortisol

Tukey HSD[a,b,c]

Grupo	N	Subset 1	2	3	4
IEA-S-D	6	1,4167			
GEA-S-cr	5	1,6000			
GEA+S-D	10	1,9000			
IEA+S-D	4	2,1250			
GEA+S-cr	6	2,3333			
IEA+S-cr	4	2,3750			
GEA-S-D	11	2,8182			
IEA-S-cr	7	4,7957			
GEA+S+D	9	6,1944	6,1944		
IEA+S+D	4	7,4375	7,4375		
IEA+S+cr	5	10,2000	10,2000	10,2000	
IEA-S+cr	6	11,9167	11,9167	11,9167	
GEA+S+cr	11	13,2273	13,2273	13,2273	
GEA-S+D	11		17,4545	17,4545	
IEA-S+D	8			22,5000	
GEA-S+cr	9				58,8889
Sig.		,092	,135	,064	1,000

Means for groups in homogeneous subsets are displayed.
Based on observed means.
The error term is Mean Square(Error) = 40,928.

a. Uses Harmonic Mean Sample Size = 6,367.

b. The group sizes are unequal. The harmonic mean of the group sizes is used. Type I error levels are not guaranteed.

c. Alpha = ,05.
