# Supplementary material for: Environmental and Pharmacological Manipulations Blunt the Stress Response of Zebrafish in a Similar Manner
Source: Sci Rep. 2016 Jun 28;6:28986. doi: 10.1038/srep28986 (PMC4924565; doi:10.1038/srep28986)
Supplement: Supplementary Information [file srep28986-s2.pdf]

```
GET
  FILE='C:\Users\Angelo\Dropbox\UFRGS HOME\ORIENTAÇÕES\Ana Giacominni-UPF\
Enriquecimento\Dados para revista\FLU data.sav'.
DATASET NAME DataSet1 WINDOW=FRONT.
UNIANOVA Cortisol BY Housing Environment Stress Drug
  /METHOD=SSTYPE(3)
  /INTERCEPT=INCLUDE
  /EMMEANS=TABLES(OVERALL)
  /PRINT=ETASQ DESCRIPTIVE
  /CRITERIA=ALPHA(.05)
  /DESIGN=Housing Environment Stress Drug Housing*Environment Housing*Stre
ss Housing*Drug Environment*Stress Environment*Drug Stress*Drug Housing*En
vironment*Stress Housing*Environment*Drug Housing*Stress*Drug Environment*
Stress*Drug
Housing*Environment*Stress*Drug.
```

Univariate Analysis of Variance

```
[DataSet1] C:\Users\Angelo\Dropbox\UFRGS HOME\ORIENTAÇÕES\Ana Giacominni-U
PF\Enriquecimento\Dados para revista\FLU data.sav
```

Between-Subjects Factors

		Value Label	N
Housing	GRUPO	GRUPO	71
	ISOLADO	ISOLADO	46
Environment	EA-	EA-	61
	EA+	EA+	56
Stress	S-	S-	54
	S+	S+	63
Drug	Flu0	Flu0	53
	Flu50	Flu50	64

Descriptive Statistics

Dependent Variable: Cortisol

Housing	Environment	Stress	Drug	Mean	Std. Deviation	N
GRUPO	EA-	S-	Flu0	1,6000	,65192	5
			Flu50	3,2500	1,65412	10
			Total	2,7000	1,59015	15
		S+	Flu0	58,8889	15,82280	9
			Flu50	16,9500	9,16348	10
			Total	36,8158	24,82170	19
		Total	Flu0	38,4286	31,07550	14
			Flu50	10,1000	9,51121	20
			Total	21,7647	25,15505	34
	EA+	S-	Flu0	2,3333	,93095	6
			Flu50	2,7500	1,33853	10
			Total	2,5938	1,18629	16
		S+	Flu0	13,2273	7,97923	11
			Flu50	11,2000	6,27694	10
			Total	12,2619	7,11621	21
		Total	Flu0	9,3824	8,29821	17
			Flu50	6,9750	6,18886	20
			Total	8,0811	7,23179	37
	Total	S-	Flu0	2,0000	,86603	11
			Flu50	3,0000	1,48678	20
			Total	2,6452	1,37352	31
		S+	Flu0	33,7750	26,11738	20
			Flu50	14,0750	8,19383	20
			Total	23,9250	21,55302	40
		Total	Flu0	22,5000	25,90560	31
			Flu50	8,5375	8,07686	40
			Total	14,6338	19,30284	71
ISOLADO	EA-	S-	Flu0	4,7957	,36395	7
			Flu50	1,9286	,67259	7
			Total	3,3621	1,57580	14
		S+	Flu0	11,9167	2,31120	6
			Flu50	12,3571	5,34411	7
			Total	12,1538	4,06911	13
		Total	Flu0	8,0823	3,99300	13
			Flu50	7,1429	6,53225	14
			Total	7,5952	5,37799	27
	EA+	S-	Flu0	2,3750	,85391	4
			Flu50	2,6000	1,29422	5
			Total	2,5000	1,06066	9

Descriptive Statistics

Dependent Variable: Cortisol

Housing	Environment	Stress	Drug	Mean	Std. Deviation	N
		S+	Flu0	10,2000	3,91472	5
			Flu50	12,4000	4,92950	5
			Total	11,3000	4,35380	10
		Total	Flu0	6,7222	4,99444	9
			Flu50	7,5000	6,18241	10
			Total	7,1316	5,50969	19
	Total	S-	Flu0	3,9155	1,33784	11
			Flu50	2,2083	,98761	12
			Total	3,0248	1,43579	23
		S+	Flu0	11,1364	3,09912	11
			Flu50	12,3750	4,94113	12
			Total	11,7826	4,11987	23
		Total	Flu0	7,5259	4,36830	22
			Flu50	7,2917	6,25355	24
			Total	7,4037	5,37651	46
Total	EA-	S-	Flu0	3,4642	1,71309	12
			Flu50	2,7059	1,46904	17
			Total	3,0197	1,59077	29
		S+	Flu0	40,1000	26,68948	15
			Flu50	15,0588	7,96061	17
			Total	26,7969	22,70671	32
		Total	Flu0	23,8174	26,99913	27
			Flu50	8,8824	8,43070	34
			Total	15,4930	20,27113	61
	EA+	S-	Flu0	2,3500	,85147	10
			Flu50	2,7000	1,27895	15
			Total	2,5600	1,12101	25
		S+	Flu0	12,2813	6,97369	16
			Flu50	11,6000	5,71089	15
			Total	11,9516	6,29729	31
		Total	Flu0	8,4615	7,32929	26
			Flu50	7,1500	6,08439	30
			Total	7,7589	6,66128	56
	Total	S-	Flu0	2,9577	1,47321	22
			Flu50	2,7031	1,36109	32
			Total	2,8069	1,39981	54
		S+	Flu0	25,7419	23,58915	31
			Flu50	13,4375	7,10719	32
			Total	19,4921	18,24707	63

Descriptive Statistics

Dependent Variable: Cortisol

Housing	Environment	Stress	Drug	Mean	Std. Deviation	N
		Total	Flu0	16,2843	21,22182	53
			Flu50	8,0703	7,41827	64
			Total	11,7912	15,76826	117

Tests of Between-Subjects Effects

Dependent Variable: Cortisol

Source	Type III Sum of Squares	df	Mean Square	F	Sig.
Corrected Model	24676,365[a]	15	1645,091	39,887	,000
Intercept	11799,947	1	11799,947	286,101	,000
Housing	1104,129	1	1104,129	26,771	,000
Environment	1235,047	1	1235,047	29,945	,000
Stress	6525,512	1	6525,512	158,217	,000
Drug	727,325	1	727,325	17,635	,000
Housing * Environment	944,751	1	944,751	22,906	,000
Housing * Stress	1260,370	1	1260,370	30,559	,000
Housing * Drug	727,209	1	727,209	17,632	,000
Environment * Stress	1101,696	1	1101,696	26,712	,000
Environment * Drug	784,969	1	784,969	19,032	,000
Stress * Drug	687,917	1	687,917	16,679	,000
Housing * Environment * Stress	1108,155	1	1108,155	26,868	,000
Housing * Environment * Drug	474,016	1	474,016	11,493	,001
Housing * Stress * Drug	1090,869	1	1090,869	26,449	,000
Environment * Stress * Drug	656,616	1	656,616	15,920	,000
Housing * Environment * Stress * Drug	747,473	1	747,473	18,123	,000
Error	4165,641	101	41,244		
Total	45108,787	117			
Corrected Total	28842,006	116			

Tests of Between-Subjects Effects

Dependent Variable: Cortisol

Source	Partial Eta Squared
Corrected Model	,856
Intercept	,739
Housing	,210
Environment	,229
Stress	,610
Drug	,149
Housing * Environment	,185
Housing * Stress	,232
Housing * Drug	,149
Environment * Stress	,209
Environment * Drug	,159
Stress * Drug	,142
Housing * Environment * Stress	,210
Housing * Environment * Drug	,102
Housing * Stress * Drug	,208
Environment * Stress * Drug	,136
Housing * Environment * Stress * Drug	,152
Error	
Total	
Corrected Total	

a. R Squared = ,856 (Adjusted R Squared = ,834)

Estimated Marginal Means

Grand Mean

Dependent Variable: Cortisol

Mean	Std. Error	95% Confidence Interval	
		Lower Bound	Upper Bound
10,548	,624	9,311	11,785

```
UNIANOVA Cortisol BY Grupo
  /METHOD=SSTYPE(3)
  /INTERCEPT=INCLUDE
  /POSTHOC=Grupo(TUKEY)
  /EMMEANS=TABLES(OVERALL)
  /PRINT=ETASQ DESCRIPTIVE
  /CRITERIA=ALPHA(.05)
  /DESIGN=Grupo.
```

Univariate Analysis of Variance

```
[DataSet1] C:\Users\Angelo\Dropbox\UFRGS HOME\ORIENTAÇÕES\Ana Giacominni-U
PF\Enriquecimento\Dados para revista\FLU data.sav
```

Between-Subjects Factors

		Value Label	N
Grupo	GEA-S-cr	GEA-S-cr	5
	GEA-S-F	GEA-S-F	10
	GEA-S+cr	GEA-S+cr	9
	GEA-S+F	GEA-S+F	10
	GEA+S-cr	GEA+S-cr	6
	GEA+S-F	GEA+S-F	10
	GEA+S+cr	GEA+S+cr	11
	GEA+S+F	GEA+S+F	10
	IEA-S-cr	IEA-S-cr	7
	IEA-S-F	IEA-S-F	7
	IEA-S+cr	IEA-S+cr	6
	IEA-S+F	IEA-S+F	7
	IEA+S-cr	IEA+S-cr	4
	IEA+S-F	IEA+S-F	5
	IEA+S+cr	IEA+S+cr	5
	IEA+S+F	IEA+S+F	5

Descriptive Statistics

Dependent Variable: Cortisol

Grupo	Mean	Std. Deviation	N
GEA-S-cr	1,6000	,65192	5
GEA-S-F	3,2500	1,65412	10
GEA-S+cr	58,8889	15,82280	9
GEA-S+F	16,9500	9,16348	10
GEA+S-cr	2,3333	,93095	6
GEA+S-F	2,7500	1,33853	10
GEA+S+cr	13,2273	7,97923	11
GEA+S+F	11,2000	6,27694	10
IEA-S-cr	4,7957	,36395	7
IEA-S-F	1,9286	,67259	7
IEA-S+cr	11,9167	2,31120	6
IEA-S+F	12,3571	5,34411	7
IEA+S-cr	2,3750	,85391	4
IEA+S-F	2,6000	1,29422	5
IEA+S+cr	10,2000	3,91472	5
IEA+S+F	12,4000	4,92950	5
Total	11,7912	15,76826	117

Tests of Between-Subjects Effects

Dependent Variable: Cortisol

Source	Type III Sum of Squares	df	Mean Square	F	Sig.	Partial Eta Squared
Corrected Model	24676,365[a]	15	1645,091	39,887	,000	,856
Intercept	11799,947	1	11799,947	286,101	,000	,739
Grupo	24676,365	15	1645,091	39,887	,000	,856
Error	4165,641	101	41,244			
Total	45108,787	117				
Corrected Total	28842,006	116				

a. R Squared = ,856 (Adjusted R Squared = ,834)

Estimated Marginal Means

Grand Mean

Dependent Variable: Cortisol

Mean	Std. Error	95% Confidence Interval	
		Lower Bound	Upper Bound
10,548	,624	9,311	11,785

Post Hoc Tests

Grupo

Multiple Comparisons

Dependent Variable: Cortisol
Tukey HSD

(I) Grupo	(J) Grupo	Mean Difference (I-J)	Std. Error	Sig.	95% Confidence Interval	
					Lower Bound	Upper Bound
GEA-S-cr	GEA-S-F	-1,6500	3,51755	1,000	-14,0110	10,7110
	GEA-S+cr	-57,2889*	3,58210	,000	-69,8767	-44,7011
	GEA-S+F	-15,3500*	3,51755	,003	-27,7110	-2,9890
	GEA+S-cr	-,7333	3,88880	1,000	-14,3989	12,9322
	GEA+S-F	-1,1500	3,51755	1,000	-13,5110	11,2110
	GEA+S+cr	-11,6273	3,46385	,078	-23,7995	,5450
	GEA+S+F	-9,6000	3,51755	,328	-21,9610	2,7610
	IEA-S-cr	-3,1957	3,76042	1,000	-16,4101	10,0187
	IEA-S-F	-,3286	3,76042	1,000	-13,5430	12,8859
	IEA-S+cr	-10,3167	3,88880	,375	-23,9822	3,3489
	IEA-S+F	-10,7571	3,76042	,254	-23,9716	2,4573
	IEA+S-cr	-,7750	4,30811	1,000	-15,9140	14,3640
	IEA+S-F	-1,0000	4,06172	1,000	-15,2732	13,2732
	IEA+S+cr	-8,6000	4,06172	,750	-22,8732	5,6732
	IEA+S+F	-10,8000	4,06172	,372	-25,0732	3,4732
GEA-S-F	GEA-S-cr	1,6500	3,51755	1,000	-10,7110	14,0110
	GEA-S+cr	-55,6389*	2,95077	,000	-66,0081	-45,2696
	GEA-S+F	-13,7000*	2,87207	,001	-23,7927	-3,6073
	GEA+S-cr	,9167	3,31638	1,000	-10,7374	12,5707
	GEA+S-F	,5000	2,87207	1,000	-9,5927	10,5927
	GEA+S+cr	-9,9773*	2,80604	,044	-19,8379	-,1166
	GEA+S+F	-7,9500	2,87207	,305	-18,0427	2,1427
	IEA-S-cr	-1,5457	3,16487	1,000	-12,6673	9,5759
	IEA-S-F	1,3214	3,16487	1,000	-9,8002	12,4430
	IEA-S+cr	-8,6667	3,31638	,402	-20,3207	2,9874
	IEA-S+F	-9,1071	3,16487	,245	-20,2287	2,0145
	IEA+S-cr	,8750	3,79939	1,000	-12,4764	14,2264
	IEA+S-F	,6500	3,51755	1,000	-11,7110	13,0110
	IEA+S+cr	-6,9500	3,51755	,832	-19,3110	5,4110
	IEA+S+F	-9,1500	3,51755	,410	-21,5110	3,2110
GEA-S+cr	GEA-S-cr	57,2889*	3,58210	,000	44,7011	69,8767
	GEA-S-F	55,6389*	2,95077	,000	45,2696	66,0081
	GEA-S+F	41,9389*	2,95077	,000	31,5696	52,3081
	GEA+S-cr	56,5556*	3,38477	,000	44,6612	68,4499
	GEA+S-F	56,1389*	2,95077	,000	45,7696	66,5081
	GEA+S+cr	45,6616*	2,88654	,000	35,5181	55,8052
	GEA+S+F	47,6889*	2,95077	,000	37,3196	58,0581
	IEA-S-cr	54,0932*	3,23646	,000	42,7200	65,4663
	IEA-S-F	56,9603*	3,23646	,000	45,5871	68,3335

Multiple Comparisons

Dependent Variable: Cortisol
Tukey HSD

(I) Grupo	(J) Grupo	Mean Difference (I-J)	Std. Error	Sig.	95% Confidence Interval	
					Lower Bound	Upper Bound
	IEA-S+cr	46,9722*	3,38477	,000	35,0779	58,8666
	IEA-S+F	46,5317*	3,23646	,000	35,1586	57,9049
	IEA+S-cr	56,5139*	3,85923	,000	42,9522	70,0755
	IEA+S-F	56,2889*	3,58210	,000	43,7011	68,8767
	IEA+S+cr	48,6889*	3,58210	,000	36,1011	61,2767
	IEA+S+F	46,4889*	3,58210	,000	33,9011	59,0767
GEA-S+F	GEA-S-cr	15,3500*	3,51755	,003	2,9890	27,7110
	GEA-S-F	13,7000*	2,87207	,001	3,6073	23,7927
	GEA-S+cr	-41,9389*	2,95077	,000	-52,3081	-31,5696
	GEA+S-cr	14,6167*	3,31638	,003	2,9626	26,2707
	GEA+S-F	14,2000*	2,87207	,000	4,1073	24,2927
	GEA+S+cr	3,7227	2,80604	,994	-6,1379	13,5834
	GEA+S+F	5,7500	2,87207	,818	-4,3427	15,8427
	IEA-S-cr	12,1543*	3,16487	,018	1,0327	23,2759
	IEA-S-F	15,0214*	3,16487	,001	3,8998	26,1430
	IEA-S+cr	5,0333	3,31638	,978	-6,6207	16,6874
	IEA-S+F	4,5929	3,16487	,985	-6,5287	15,7145
	IEA+S-cr	14,5750*	3,79939	,019	1,2236	27,9264
	IEA+S-F	14,3500*	3,51755	,008	1,9890	26,7110
	IEA+S+cr	6,7500	3,51755	,861	-5,6110	19,1110
	IEA+S+F	4,5500	3,51755	,995	-7,8110	16,9110
GEA+S-cr	GEA-S-cr	,7333	3,88880	1,000	-12,9322	14,3989
	GEA-S-F	-,9167	3,31638	1,000	-12,5707	10,7374
	GEA-S+cr	-56,5556*	3,38477	,000	-68,4499	-44,6612
	GEA-S+F	-14,6167*	3,31638	,003	-26,2707	-2,9626
	GEA+S-F	-,4167	3,31638	1,000	-12,0707	11,2374
	GEA+S+cr	-10,8939	3,25936	,081	-22,3476	,5597
	GEA+S+F	-8,8667	3,31638	,362	-20,5207	2,7874
	IEA-S-cr	-2,4624	3,57295	1,000	-15,0180	10,0933
	IEA-S-F	,4048	3,57295	1,000	-12,1509	12,9604
	IEA-S+cr	-9,5833	3,70783	,421	-22,6129	3,4463
	IEA-S+F	-10,0238	3,57295	,283	-22,5795	2,5318
	IEA+S-cr	-,0417	4,14548	1,000	-14,6092	14,5259
	IEA+S-F	-,2667	3,88880	1,000	-13,9322	13,3989
	IEA+S+cr	-7,8667	3,88880	,807	-21,5322	5,7989
	IEA+S+F	-10,0667	3,88880	,418	-23,7322	3,5989
GEA+S-F	GEA-S-cr	1,1500	3,51755	1,000	-11,2110	13,5110
	GEA-S-F	-,5000	2,87207	1,000	-10,5927	9,5927
	GEA-S+cr	-56,1389*	2,95077	,000	-66,5081	-45,7696

Multiple Comparisons

Dependent Variable: Cortisol
Tukey HSD

(I) Grupo	(J) Grupo	Mean Difference (I-J)	Std. Error	Sig.	95% Confidence Interval	
					Lower Bound	Upper Bound
	GEA-S+F	-14,2000*	2,87207	,000	-24,2927	-4,1073
	GEA+S-cr	,4167	3,31638	1,000	-11,2374	12,0707
	GEA+S+cr	-10,4773*	2,80604	,026	-20,3379	-,6166
	GEA+S+F	-8,4500	2,87207	,214	-18,5427	1,6427
	IEA-S-cr	-2,0457	3,16487	1,000	-13,1673	9,0759
	IEA-S-F	,8214	3,16487	1,000	-10,3002	11,9430
	IEA-S+cr	-9,1667	3,31638	,307	-20,8207	2,4874
	IEA-S+F	-9,6071	3,16487	,173	-20,7287	1,5145
	IEA+S-cr	,3750	3,79939	1,000	-12,9764	13,7264
	IEA+S-F	,1500	3,51755	1,000	-12,2110	12,5110
	IEA+S+cr	-7,4500	3,51755	,749	-19,8110	4,9110
	IEA+S+F	-9,6500	3,51755	,319	-22,0110	2,7110
GEA+S+cr	GEA-S-cr	11,6273	3,46385	,078	-,5450	23,7995
	GEA-S-F	9,9773*	2,80604	,044	,1166	19,8379
	GEA-S+cr	-45,6616*	2,88654	,000	-55,8052	-35,5181
	GEA-S+F	-3,7227	2,80604	,994	-13,5834	6,1379
	GEA+S-cr	10,8939	3,25936	,081	-,5597	22,3476
	GEA+S-F	10,4773*	2,80604	,026	,6166	20,3379
	GEA+S+F	2,0273	2,80604	1,000	-7,8334	11,8879
	IEA-S-cr	8,4316	3,10507	,336	-2,4799	19,3430
	IEA-S-F	11,2987*	3,10507	,035	,3872	22,2102
	IEA-S+cr	1,3106	3,25936	1,000	-10,1431	12,7643
	IEA-S+F	,8701	3,10507	1,000	-10,0413	11,7816
	IEA+S-cr	10,8523	3,74973	,237	-2,3246	24,0291
	IEA+S-F	10,6273	3,46385	,161	-1,5450	22,7995
	IEA+S+cr	3,0273	3,46385	1,000	-9,1450	15,1995
	IEA+S+F	,8273	3,46385	1,000	-11,3450	12,9995
GEA+S+F	GEA-S-cr	9,6000	3,51755	,328	-2,7610	21,9610
	GEA-S-F	7,9500	2,87207	,305	-2,1427	18,0427
	GEA-S+cr	-47,6889*	2,95077	,000	-58,0581	-37,3196
	GEA-S+F	-5,7500	2,87207	,818	-15,8427	4,3427
	GEA+S-cr	8,8667	3,31638	,362	-2,7874	20,5207
	GEA+S-F	8,4500	2,87207	,214	-1,6427	18,5427
	GEA+S+cr	-2,0273	2,80604	1,000	-11,8879	7,8334
	IEA-S-cr	6,4043	3,16487	,806	-4,7173	17,5259
	IEA-S-F	9,2714	3,16487	,219	-1,8502	20,3930
	IEA-S+cr	-,7167	3,31638	1,000	-12,3707	10,9374
	IEA-S+F	-1,1571	3,16487	1,000	-12,2787	9,9645
	IEA+S-cr	8,8250	3,79939	,608	-4,5264	22,1764

Multiple Comparisons

Dependent Variable: Cortisol
Tukey HSD

(I) Grupo	(J) Grupo	Mean Difference (I-J)	Std. Error	Sig.	95% Confidence Interval Lower Bound	Upper Bound
	IEA+S-F	8,6000	3,51755	,520	-3,7610	20,9610
	IEA+S+cr	1,0000	3,51755	1,000	-11,3610	13,3610
	IEA+S+F	-1,2000	3,51755	1,000	-13,5610	11,1610
IEA-S-cr	GEA-S-cr	3,1957	3,76042	1,000	-10,0187	16,4101
	GEA-S-F	1,5457	3,16487	1,000	-9,5759	12,6673
	GEA-S+cr	-54,0932*	3,23646	,000	-65,4663	-42,7200
	GEA-S+F	-12,1543*	3,16487	,018	-23,2759	-1,0327
	GEA+S-cr	2,4624	3,57295	1,000	-10,0933	15,0180
	GEA+S-F	2,0457	3,16487	1,000	-9,0759	13,1673
	GEA+S+cr	-8,4316	3,10507	,336	-19,3430	2,4799
	GEA+S+F	-6,4043	3,16487	,806	-17,5259	4,7173
	IEA-S-F	2,8671	3,43278	1,000	-9,1959	14,9302
	IEA-S+cr	-7,1210	3,57295	,823	-19,6766	5,4347
	IEA-S+F	-7,5614	3,43278	,693	-19,6245	4,5016
	IEA+S-cr	2,4207	4,02529	1,000	-11,7245	16,5659
	IEA+S-F	2,1957	3,76042	1,000	-11,0187	15,4101
	IEA+S+cr	-5,4043	3,76042	,987	-18,6187	7,8101
	IEA+S+F	-7,6043	3,76042	,807	-20,8187	5,6101
IEA-S-F	GEA-S-cr	,3286	3,76042	1,000	-12,8859	13,5430
	GEA-S-F	-1,3214	3,16487	1,000	-12,4430	9,8002
	GEA-S+cr	-56,9603*	3,23646	,000	-68,3335	-45,5871
	GEA-S+F	-15,0214*	3,16487	,001	-26,1430	-3,8998
	GEA+S-cr	-,4048	3,57295	1,000	-12,9604	12,1509
	GEA+S-F	-,8214	3,16487	1,000	-11,9430	10,3002
	GEA+S+cr	-11,2987*	3,10507	,035	-22,2102	-,3872
	GEA+S+F	-9,2714	3,16487	,219	-20,3930	1,8502
	IEA-S-cr	-2,8671	3,43278	1,000	-14,9302	9,1959
	IEA-S+cr	-9,9881	3,57295	,289	-22,5437	2,5675
	IEA-S+F	-10,4286	3,43278	,172	-22,4916	1,6345
	IEA+S-cr	-,4464	4,02529	1,000	-14,5916	13,6988
	IEA+S-F	-,6714	3,76042	1,000	-13,8859	12,5430
	IEA+S+cr	-8,2714	3,76042	,695	-21,4859	4,9430
	IEA+S+F	-10,4714	3,76042	,295	-23,6859	2,7430
IEA-S+cr	GEA-S-cr	10,3167	3,88880	,375	-3,3489	23,9822
	GEA-S-F	8,6667	3,31638	,402	-2,9874	20,3207
	GEA-S+cr	-46,9722*	3,38477	,000	-58,8666	-35,0779
	GEA-S+F	-5,0333	3,31638	,978	-16,6874	6,6207
	GEA+S-cr	9,5833	3,70783	,421	-3,4463	22,6129
	GEA+S-F	9,1667	3,31638	,307	-2,4874	20,8207

Multiple Comparisons

Dependent Variable: Cortisol
Tukey HSD

(I) Grupo	(J) Grupo	Mean Difference (I-J)	Std. Error	Sig.	95% Confidence Interval	
					Lower Bound	Upper Bound
	GEA+S+cr	-1,3106	3,25936	1,000	-12,7643	10,1431
	GEA+S+F	,7167	3,31638	1,000	-10,9374	12,3707
	IEA-S-cr	7,1210	3,57295	,823	-5,4347	19,6766
	IEA-S-F	9,9881	3,57295	,289	-2,5675	22,5437
	IEA-S+F	-,4405	3,57295	1,000	-12,9961	12,1152
	IEA+S-cr	9,5417	4,14548	,623	-5,0259	24,1092
	IEA+S-F	9,3167	3,88880	,555	-4,3489	22,9822
	IEA+S+cr	1,7167	3,88880	1,000	-11,9489	15,3822
	IEA+S+F	-,4833	3,88880	1,000	-14,1489	13,1822
IEA-S+F	GEA-S-cr	10,7571	3,76042	,254	-2,4573	23,9716
	GEA-S-F	9,1071	3,16487	,245	-2,0145	20,2287
	GEA-S+cr	-46,5317*	3,23646	,000	-57,9049	-35,1586
	GEA-S+F	-4,5929	3,16487	,985	-15,7145	6,5287
	GEA+S-cr	10,0238	3,57295	,283	-2,5318	22,5795
	GEA+S-F	9,6071	3,16487	,173	-1,5145	20,7287
	GEA+S+cr	-,8701	3,10507	1,000	-11,7816	10,0413
	GEA+S+F	1,1571	3,16487	1,000	-9,9645	12,2787
	IEA-S-cr	7,5614	3,43278	,693	-4,5016	19,6245
	IEA-S-F	10,4286	3,43278	,172	-1,6345	22,4916
	IEA-S+cr	,4405	3,57295	1,000	-12,1152	12,9961
	IEA+S-cr	9,9821	4,02529	,494	-4,1631	24,1274
	IEA+S-F	9,7571	3,76042	,414	-3,4573	22,9716
	IEA+S+cr	2,1571	3,76042	1,000	-11,0573	15,3716
	IEA+S+F	-,0429	3,76042	1,000	-13,2573	13,1716
IEA+S-cr	GEA-S-cr	,7750	4,30811	1,000	-14,3640	15,9140
	GEA-S-F	-,8750	3,79939	1,000	-14,2264	12,4764
	GEA-S+cr	-56,5139*	3,85923	,000	-70,0755	-42,9522
	GEA-S+F	-14,5750*	3,79939	,019	-27,9264	-1,2236
	GEA+S-cr	,0417	4,14548	1,000	-14,5259	14,6092
	GEA+S-F	-,3750	3,79939	1,000	-13,7264	12,9764
	GEA+S+cr	-10,8523	3,74973	,237	-24,0291	2,3246
	GEA+S+F	-8,8250	3,79939	,608	-22,1764	4,5264
	IEA-S-cr	-2,4207	4,02529	1,000	-16,5659	11,7245
	IEA-S-F	,4464	4,02529	1,000	-13,6988	14,5916
	IEA-S+cr	-9,5417	4,14548	,623	-24,1092	5,0259
	IEA-S+F	-9,9821	4,02529	,494	-24,1274	4,1631
	IEA+S-F	-,2250	4,30811	1,000	-15,3640	14,9140
	IEA+S+cr	-7,8250	4,30811	,905	-22,9640	7,3140
	IEA+S+F	-10,0250	4,30811	,605	-25,1640	5,1140

Multiple Comparisons

Dependent Variable: Cortisol
Tukey HSD

(I) Grupo	(J) Grupo	Mean Difference (I-J)	Std. Error	Sig.	95% Confidence Interval	
					Lower Bound	Upper Bound
IEA+S-F	GEA-S-cr	1,0000	4,06172	1,000	-13,2732	15,2732
	GEA-S-F	-,6500	3,51755	1,000	-13,0110	11,7110
	GEA-S+cr	-56,2889*	3,58210	,000	-68,8767	-43,7011
	GEA-S+F	-14,3500*	3,51755	,008	-26,7110	-1,9890
	GEA+S-cr	,2667	3,88880	1,000	-13,3989	13,9322
	GEA+S-F	-,1500	3,51755	1,000	-12,5110	12,2110
	GEA+S+cr	-10,6273	3,46385	,161	-22,7995	1,5450
	GEA+S+F	-8,6000	3,51755	,520	-20,9610	3,7610
	IEA-S-cr	-2,1957	3,76042	1,000	-15,4101	11,0187
	IEA-S-F	,6714	3,76042	1,000	-12,5430	13,8859
	IEA-S+cr	-9,3167	3,88880	,555	-22,9822	4,3489
	IEA-S+F	-9,7571	3,76042	,414	-22,9716	3,4573
	IEA+S-cr	,2250	4,30811	1,000	-14,9140	15,3640
	IEA+S+cr	-7,6000	4,06172	,883	-21,8732	6,6732
	IEA+S+F	-9,8000	4,06172	,543	-24,0732	4,4732
IEA+S+cr	GEA-S-cr	8,6000	4,06172	,750	-5,6732	22,8732
	GEA-S-F	6,9500	3,51755	,832	-5,4110	19,3110
	GEA-S+cr	-48,6889*	3,58210	,000	-61,2767	-36,1011
	GEA-S+F	-6,7500	3,51755	,861	-19,1110	5,6110
	GEA+S-cr	7,8667	3,88880	,807	-5,7989	21,5322
	GEA+S-F	7,4500	3,51755	,749	-4,9110	19,8110
	GEA+S+cr	-3,0273	3,46385	1,000	-15,1995	9,1450
	GEA+S+F	-1,0000	3,51755	1,000	-13,3610	11,3610
	IEA-S-cr	5,4043	3,76042	,987	-7,8101	18,6187
	IEA-S-F	8,2714	3,76042	,695	-4,9430	21,4859
	IEA-S+cr	-1,7167	3,88880	1,000	-15,3822	11,9489
	IEA-S+F	-2,1571	3,76042	1,000	-15,3716	11,0573
	IEA+S-cr	7,8250	4,30811	,905	-7,3140	22,9640
	IEA+S-F	7,6000	4,06172	,883	-6,6732	21,8732
	IEA+S+F	-2,2000	4,06172	1,000	-16,4732	12,0732
IEA+S+F	GEA-S-cr	10,8000	4,06172	,372	-3,4732	25,0732
	GEA-S-F	9,1500	3,51755	,410	-3,2110	21,5110
	GEA-S+cr	-46,4889*	3,58210	,000	-59,0767	-33,9011
	GEA-S+F	-4,5500	3,51755	,995	-16,9110	7,8110
	GEA+S-cr	10,0667	3,88880	,418	-3,5989	23,7322
	GEA+S-F	9,6500	3,51755	,319	-2,7110	22,0110
	GEA+S+cr	-,8273	3,46385	1,000	-12,9995	11,3450
	GEA+S+F	1,2000	3,51755	1,000	-11,1610	13,5610
	IEA-S-cr	7,6043	3,76042	,807	-5,6101	20,8187

Multiple Comparisons

Dependent Variable: Cortisol
Tukey HSD

(I) Grupo	(J) Grupo	Mean Difference (I-J)	Std. Error	Sig.	95% Confidence Interval Lower Bound	Upper Bound
	IEA-S-F	10,4714	3,76042	,295	-2,7430	23,6859
	IEA-S+cr	,4833	3,88880	1,000	-13,1822	14,1489
	IEA-S+F	,0429	3,76042	1,000	-13,1716	13,2573
	IEA+S-cr	10,0250	4,30811	,605	-5,1140	25,1640
	IEA+S-F	9,8000	4,06172	,543	-4,4732	24,0732
	IEA+S+cr	2,2000	4,06172	1,000	-12,0732	16,4732

Dependent Variable: Cortisol
Tukey HSD

Homogeneous Subsets

Cortisol

Tukey HSD[a,b,c]

Grupo	N	Subset 1	2	3
GEA-S-cr	5	1,6000		
IEA-S-F	7	1,9286		
GEA+S-cr	6	2,3333		
IEA+S-cr	4	2,3750		
IEA+S-F	5	2,6000		
GEA+S-F	10	2,7500		
GEA-S-F	10	3,2500		
IEA-S-cr	7	4,7957	4,7957	
IEA+S+cr	5	10,2000	10,2000	
GEA+S+F	10	11,2000	11,2000	
IEA-S+cr	6	11,9167	11,9167	
IEA-S+F	7	12,3571	12,3571	
IEA+S+F	5	12,4000	12,4000	
GEA+S+cr	11	13,2273	13,2273	
GEA-S+F	10		16,9500	
GEA-S+cr	9			58,8889
Sig.		,091	,061	1,000

Means for groups in homogeneous subsets are displayed.
Based on observed means.
The error term is Mean Square(Error) = 41,244.

a. Uses Harmonic Mean Sample Size = 6,628.

b. The group sizes are unequal. The harmonic mean of the group sizes is used. Type I error levels are

b. The group sizes are unequal. The harmonic mean of the group sizes is used. Type I error levels are not guaranteed.

c. Alpha = ,05.
